# Diagnostic accuracy of the Cepheid MTB host response assay for the detection of pulmonary TB

**DOI:** 10.5588/ijtldopen.25.0380

**Published:** 2025-11-12

**Authors:** Y. Alkabab, Y. Rani, M. Farhad, C. Zamudio, T. Caceres, H. Cox, W. Dowling, E. Nakabugo, L. Nakiyingi, C. Hoang, N. Van Hung, H. Lipson, H. Jinna, D.T. Armstrong, A. Borkman, M. de Vos, S. Kim, S.E. Dorman, A.P. Nicholson, the FEND-TB Consortium

**Affiliations:** 1 Medical University of South Carolina, Charleston, SC, USA;; 2 Frontier Science Foundation, Brookline, MA, USA;; 3 Universidad Peruana Cayetano Heredia, Lima, Peru;; 4 University of Cape Town, Cape Town, South Africa;; 5 Makerere University, Kampala, Uganda;; 6 National Lung Hospital, Hanoi, Vietnam;; 7 Rutgers New Jersey Medical School, Newark, NJ, USA;; 8 Johns Hopkins University, Baltimore, MD, USA;; 9 Foundation for Innovative New Diagnostics, Geneva, Switzerland.

**Keywords:** tuberculosis, *Mycobacterium tuberculosis*, host response, diagnostic accuracy, sensitivity, specificity

## Abstract

**BACKGROUND:**

Current sputum-based diagnostic approaches fail to identify some individuals with active TB, underscoring the need for tests that do not rely on sputum. This study assessed the diagnostic accuracy of the Xpert MTB Host Response (Xpert HR) blood test for detecting pulmonary TB.

**METHODS:**

We conducted a prospective study in adults with pulmonary TB symptoms who underwent Xpert HR testing. Diagnostic accuracy was assessed against a microbiological reference standard of sputum cultures and molecular testing.

**RESULTS:**

Among 813 participants, 52% were female, median age was 38 years, and 192 (24%) were HIV-positive. For Xpert HR performed using capillary blood, sensitivity was 91% (95% confidence interval [CI]: 86–94), specificity was 45% (95% CI: 41–49), and area under the receiver operating characteristic curve was 0.85 (95% CI: 0.81–0.89). Country-specific differences in performance were observed. Sensitivity was 93% (95% CI: 76–98) and specificity was 18% (95% CI: 13–27) among people living with HIV.

**CONCLUSION:**

The Xpert HR TB assay demonstrated promising sensitivity, but its specificity did not meet minimum target product profiles for a TB triage or case detection test. In its current form, Xpert HR cannot replace more sensitive nucleic acid amplification tests in sputum-productive individuals.

Accurate, rapid TB diagnosis is essential for optimising patient outcomes and reducing transmission. Despite current methods for detecting *Mycobacterium tuberculosis* (MTB) in sputum, a substantial diagnostic gap remains. In 2023, approximately 30%–40% of the 10.8 million people with active TB were undiagnosed.^1–3^ Triage tests with high negative predictive value (NPV) could help narrow this gap by definitively ruling out TB in the TB-symptomatic individuals presenting for care.^4^ The World Health Organization (WHO) has issued target product profiles (TPPs) to guide TB triage test development, advocating for non-sputum-based testing, operational characteristics that allow for use in peripheral health settings, and recommended minimum sensitivity and specificity targets of 90% and 70%, respectively.^5^ Transcriptomics studies using blood have identified human transcriptional signatures associated with TB disease, enabling differentiation between TB disease and other conditions.^6–8^ A blood-based three-gene mRNA signature has been validated in several cohorts and incorporated into a cartridge-based automated quantitative polymerase chain reaction test (Xpert MTB Host Response [Xpert HR]) that uses the GeneXpert platform (Cepheid, Sunnyvale, CA, USA).^9^ Test output is a numeric TB score calculated from cycle threshold (CT) values of the target genes *GBP5*, *DUSP3*, and *TBP*. The Xpert HR test accommodates fingerstick capillary blood samples and can deliver results within 1 h of test initiation.

We conducted a prospective study of the Xpert HR test to detect pulmonary TB, aiming to evaluate its performance overall and in key subgroups, and compared test performance using capillary versus venous blood.

## METHODS

This prospective diagnostic accuracy study was conducted among adults (≥18 years) with signs or symptoms suggestive of pulmonary TB presenting to outpatient and inpatient settings in Peru, South Africa, Uganda, and Vietnam as part of the *Feasibility of New Diagnostics for TB* (FEND-TB) study. Individuals who had received anti-TB treatment within 6 months preceding enrolment were excluded.

### Clinical procedures

Demographic, medical history, and symptom data were collected using a standardised form. Three spontaneously expectorated sputum specimens were collected over 2 days for microbiological reference standard (MRS) tests. HIV testing was performed unless documentation of test results within the past 3 months was available.

For investigational testing using the Xpert HR cartridge, capillary blood (via fingerprick) was collected at all four sites; additional venous blood was collected for a subset of consecutive participants in Uganda and Vietnam. Fingerprick blood was collected with a BD Microtainer lancet (Becton, Dickinson and Company, Franklin Lakes, NJ, USA) and transferred into a minivet containing K3 EDTA. Venous blood was also collected in K3 EDTA tubes.

### Laboratory procedures

#### Index tests

**Figure 1.**
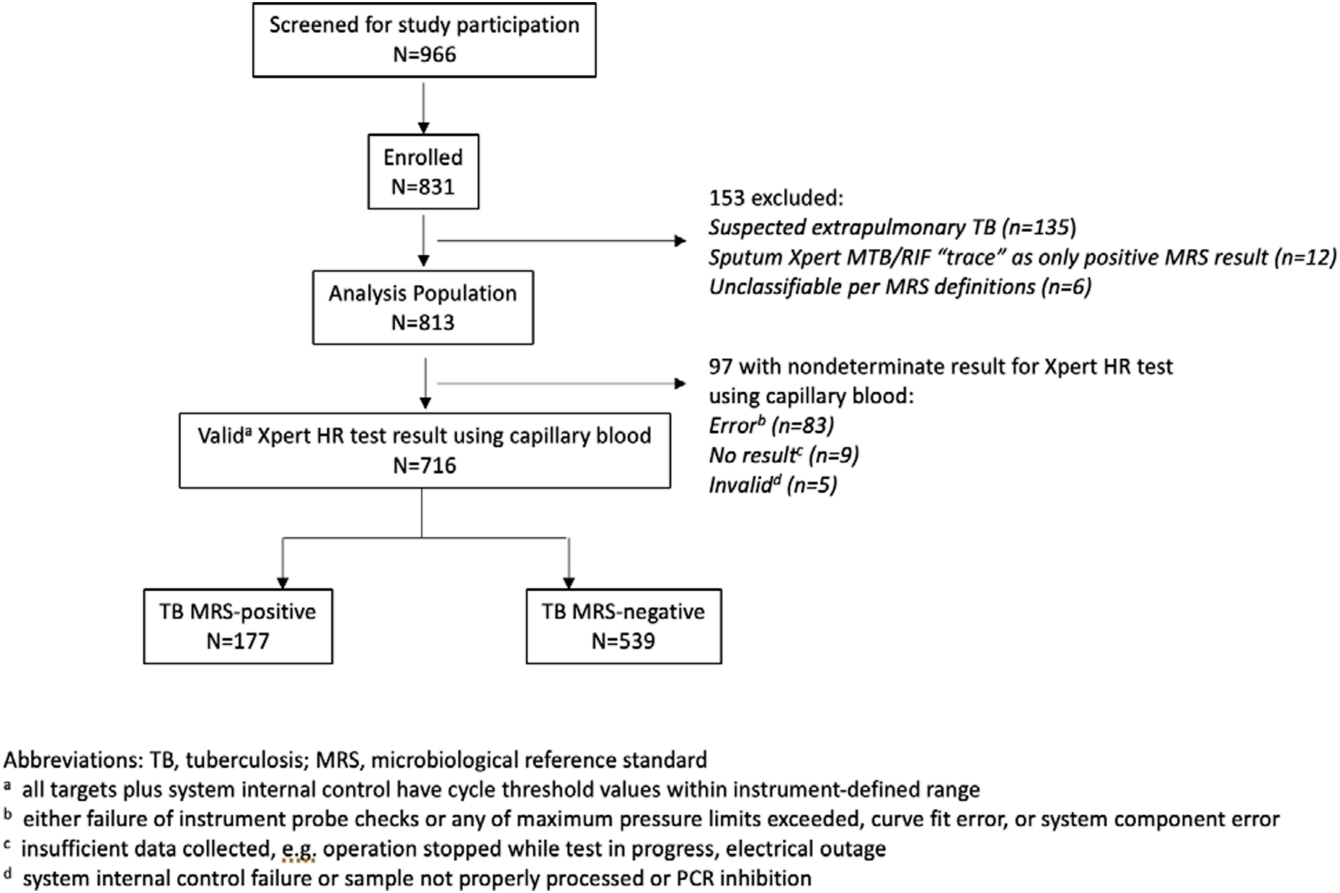
Participant flow diagram.

The main index test was the Xpert HR assay performed using capillary blood, with testing initiated within 1 h of blood collection by transferring 100 µL of blood from the minivet to the Xpert HR cartridge. For venous blood, the collection tube was briefly vortexed, and a 100 µL aliquot was removed and tested using Xpert HR within 1 h of collection. The venous blood collection tube was then refrigerated (2°C–8°C) for 24 h, vortexed briefly, and a second 100 µL aliquot was tested. Per manufacturer’s guidance, test results were classified as valid, invalid, error, or no result (Figure 1). For valid results, the instrument-generated TB score incorporating *GBP5*, *DUSP3*, and *TBP* CTs was recorded.

#### Reference standard tests

Day 1 and Day 2 sputum specimens were tested immediately after collection using the Mycobacterial Growth Indicator System (MGIT, BD Microbiology Systems, Sparks, MD, USA), Löwenstein-Jensen (LJ) solid medium,^10^ Xpert MTB/RIF Ultra, and smear microscopy. Cultures positive for the growth of acid-fast bacilli underwent confirmation of *Mycobacterium tuberculosis* complex (MTBC) by MPT64/MPB64 antigen detection (Bruker-Hain Lifescience, Nehren, Germany).^11^ Culture results were classified as MTB detected, MTB not detected, or contaminated.

This study used an MRS based on MGIT culture, LJ culture, and Xpert MTB/RIF Ultra results from the Day 1 and Day 2 sputum specimens. Participants were classified as MRS-positive (MRS+) based on detection of MTBC in at least one sputum specimen using at least one of these three test platforms. Among participants without a positive result for MTBC, those with at least two negative test results were classified as MRS-negative (MRS−). Participants not meeting the definitions of MRS+ or MRS− and those in whom the only positive sputum result was semi-quantitative result of ‘trace’ using Xpert MTB/RIF Ultra were considered unclassifiable. MRS+ participants were subclassified as sputum smear microscopy–positive if they had at least one positive result and classified as sputum smear microscopy–negative if they had no positive result and at least one negative result. The classification was performed programmatically by a statistician and did not include a review of clinical data.

### Statistical analysis

Descriptive statistics summarised participant characteristics by country; categorical variables were reported as frequencies and percentages, and continuous variables were summarised using medians and interquartile ranges. *P* values are provided using Fisher’s exact test for nominal variables, the Jonckheere–Terpstra test for ordinal variables, and Wilcoxon’s or the Kruskal–Wallis test for continuous variables. Participants with non-valid Xpert HR results were excluded from analysis of CT values, TB scores, and diagnostic accuracy. Using the MRS, diagnostic accuracy of the Xpert HR test was evaluated by sensitivity, specificity, observed positive predictive value (PPV), and observed NPV, with corresponding 95% confidence intervals (CIs). Unadjusted CIs were calculated using the Wilson score method, while adjusted estimates used stratified CIs to adjust for country.^12^ Weights in the stratified analysis were proportional to the number of participants from each country within the MRS category. Subgroup analyses were performed based on country, HIV status, age category, and diabetes mellitus (DM). For estimation of sensitivity, specificity, PPV, and NPV, we used as the cut-off the capillary blood TB score that achieved at least 90% sensitivity and maximised specificity for the overall group. Receiver operating characteristic (ROC) curves and the corresponding area under the ROC curve (AUC) with 95% CIs were used to describe the trade-off between sensitivity and specificity. Differences in AUCs between pairs of sample types were calculated and compared using Delong’s test, which accounts for the correlation of test results within a participant. Bland–Altman analysis and Passing and Bablok regression were used to compare the TB scores between the capillary and the venous sample types. ROC curves were constructed for individual genes to evaluate their contributions to overall diagnostic performance. Histograms and smoothed kernel density estimates were used to visualise differences between MRS+ and MRS− groups. Missing data were assumed to be missing completely at random. All statistical analyses were performed using SAS 9.4 (SAS Institute Inc., Cary, NC, USA) and R 3.6.0 (The R Foundation for Statistical Computing) using pROC^13^ and ggplot2^14^ packages. All tests were two-sided; statistical significance was set at 5%, though focus remained on descriptive accuracy and estimation. Study reporting follows the *Standards for Reporting of Diagnostic Accuracy Studies*.^15^

### Ethical statement

The study protocol and activities were approved by the Rutgers New Jersey Medical School Institutional Review Board and by ethics committees in participating countries. Participants provided written informed consent.

## RESULTS

Between September 2022 and January 2024, 966 people were screened (Figure 1). 135 (14%) were suspected to have extra-pulmonary TB and were excluded. Among the 831 enrolled individuals, 18 (2%) were excluded from analyses – 6 were unclassifiable based on the MRS, and for 12 MRS+ participants, the only MTBC-positive result was a sputum Xpert MTB/RIF Ultra test with ‘trace’ semi-quantitative result. Among the 813 participants, 9% were enrolled from inpatient settings, the median (interquartile range [IQR]) age was 38 years (28, 50), 422 (52%) were female, and 192 (24%) were people living with HIV (PLWH) (Table 1). The majority (96%) of PLWH were enrolled from South Africa and Uganda. A total of 196 (24%) participants were classified as MRS+. Among participants classified as MRS+, 170 (87%) were positive by both culture and Xpert MTB/RIF, 12 (6%) were culture-only positive, and 14 (7%) were Xpert-only positive.

**Table 1.** Participant characteristics, overall and by country, for the analysis population.

Characteristic, N (%)	Country				Total, N = 813
	Peru, N = 235	South Africa, N = 337	Uganda, N = 148	Vietnam, N = 93	
Enrolment setting					
Inpatient	0 (0%)	0 (0%)	33 (22%)	40 (43%)	73 (9%)
Outpatient	235 (100%)	337 (100%)	115 (78%)	53 (57%)	740 (91%)
Female sex	120 (51%)	204 (61%)	59 (40%)	39 (42%)	422 (52%)
Age, years: median (IQR)	37 (27, 51)	36 (27, 46)	35 (27, 44)	51 (37, 61)	38 (28, 50)
Race					
African/Black	0 (0%)	337 (100%)	148 (100%)	0 (0%)	485 (60%)
Asian	0 (0%)	0 (0%)	0 (0%)	93 (100%)	93 (11%)
Unknown/not reported	235 (100%)	0 (0%)	0 (0%)	0 (0%)	235 (29%)
Ethnicity					
Hispanic or Latino	235 (100%)	0 (0%)	1 (1%)	0 (0%)	236 (29%)
Not Hispanic or Latino	0 (0%)	337 (100%)	147 (99%)	87 (94%)	571 (70%)
Unknown/not reported	0 (0%)	0 (0%)	0 (0%)	6 (6%)	6 (1%)
Cough for 2 weeks or more	235 (100%)	337 (100%)	148 (100%)	92 (99%)	812 (100%)
Fever	126 (54%)	231 (69%)	89 (60%)	26 (28%)	472 (58%)
Night sweats	185 (79%)	273 (81%)	59 (40%)	12 (13%)	529 (65%)
Weight loss	151 (64%)	199 (59%)	86 (58%)	30 (32%)	466 (57%)
Previous TB diagnosis	57 (24%)	66 (20%)	26 (18%)	4 (4%)	153 (19%)
Living with HIV	7 (3%)	125 (37%)	60 (41%)	0 (0%)	192 (24%)
CD4, cells/µL: median (IQR)^**A**^	173 (60, 227)	357 (233, 646)	348 (183, 703)	NA	348 (207, 664)
Diabetes^**B**^	14 (6%)	13 (4%)	4 (3%)	9 (10%)	40 (5%)
Venous blood collected	0 (0%)	0 (0%)	42 (28%)	93 (100%)	133 (100%)
TB MRS status					
MRS-positive	70 (30%)	57 (17%)	32 (22%)	37 (40%)	196 (24%)
MRS-negative	165 (70%)	280 (83%)	116 (78%)	56 (60%)	617 (76%)
Sputum AFB smear status/grade^**C**^					
Negative	173 (74%)	300 (89%)	119 (81%)	77 (83%)	669 (82%)
Scanty	22 (9%)	6 (2%)	0 (0%)	1 (1%)	29 (4%)
1+	19 (8%)	3 (1%)	4 (3%)	8 (9%)	34 (4%)
2+	11 (5%)	8 (2%)	10 (7%)	3 (3%)	32 (4%)
3+	10 (4%)	20 (6%)	12 (8%)	4 (4%)	46 (6%)
Positive, not quantified	0 (0%)	0 (0%)	2 (1%)	0 (0%)	2 (<1%)
Sputum Xpert MTB/RIF Ultra					
Positive	66 (28%)	53 (16%)	30 (20%)	35 (38%)	184 (23%)
Negative	169 (72%)	282 (84%)	117 (79%)	58 (62%)	626 (77%)
Invalid test	0 (0%)	1 (<1%)	0 (0%)	0 (0%)	1 (<1%)
Unknown	0 (0%)	1 (<1%)	1 (<1%)	0 (0%)	2 (<1%)

MRS = microbiological reference standard; IQR = interquartile range (i.e., 25th and 75th percentiles); NA = not applicable.

A CD4 count is provided among people living with HIV; missing CD4 count for four and one people living with HIV in South Africa and Uganda, respectively.

B Diabetes status was obtained by participant history and chart review.

C Sputum AFB smear status was not available for one participant and not quantified for two participants from Uganda.

### Results for capillary blood tested on Xpert HR

Non-determinate results: among the 813 participants, 716 (88%) had a valid Xpert HR test result; the Xpert HR test result was classified as error for 83 (10%), no result for 9 (1%), and invalid for 5 (<1%) (Figure 1).

**Figure 2.**
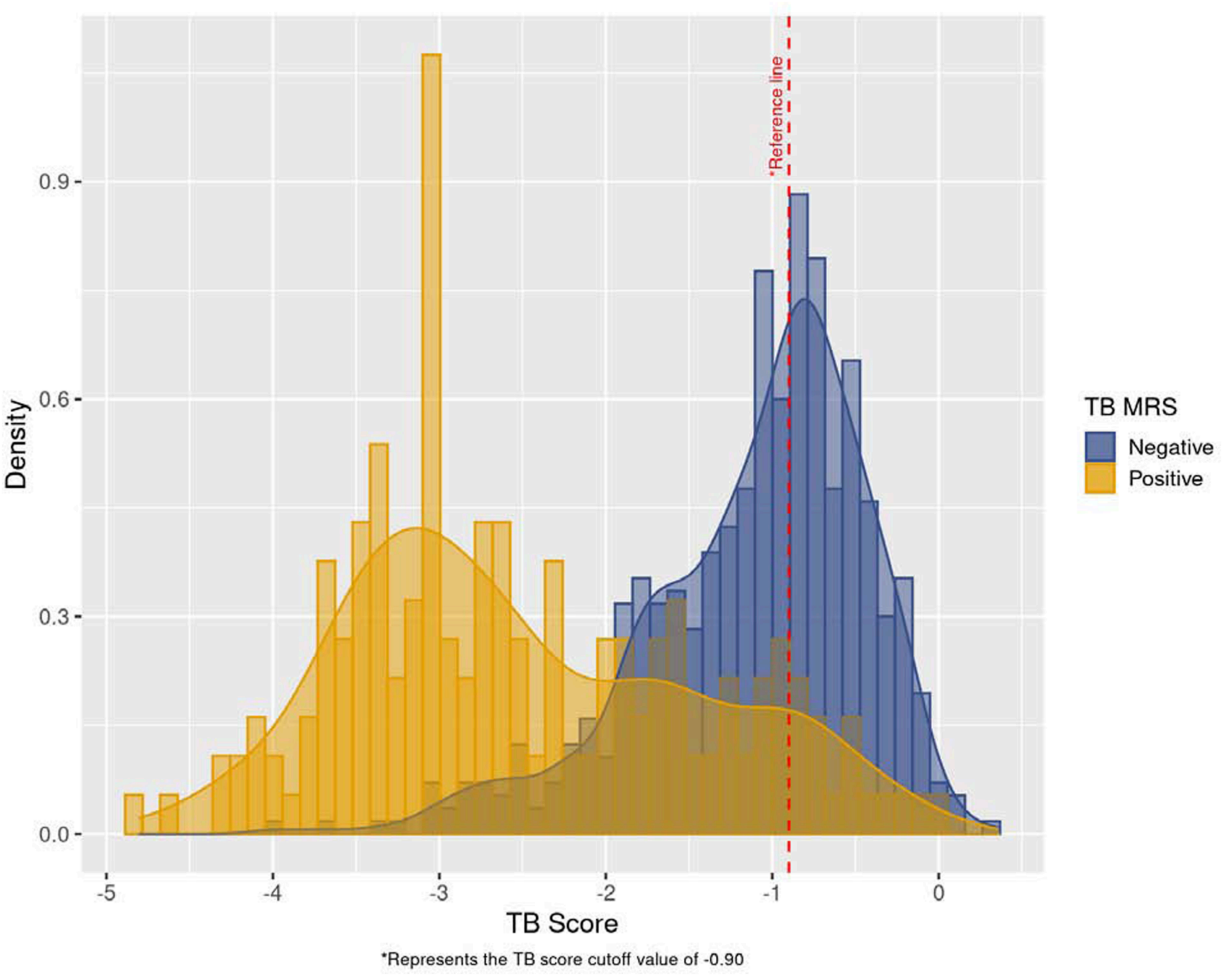
Histogram of Xpert HR TB scores for MRS-positive participants and MRS-negative participants. MRS = microbiological reference standard; HR = host response.

TB scores: the median TB score for capillary blood was −1.2 (IQR −1.9, −0.8) (Supplementary Data Table). The distribution of TB scores was lower for MRS+ versus MRS−; the median TB score for MRS− participants was −1.0 (IQR −1.5, −0.7), and for MRS+ participants was −2.7 (IQR −3.4, −1.7) (Figure 2 and Supplementary Data Table). Among MRS+ participants, TB scores were inversely associated with sputum bacillary burden as assessed by sputum smear microscopy and semi-quantitative Xpert MTB/RIF Ultra (Supplementary Data Table). There was significant variation in median TB scores by country (*P* = 0.004). Median TB scores were lower in Vietnam than in the other study settings. Median TB scores were not different by diabetes status (*P* = 0.26) or sex (*P* = 0.38) (Supplementary Data Table).

**Figure 3.**
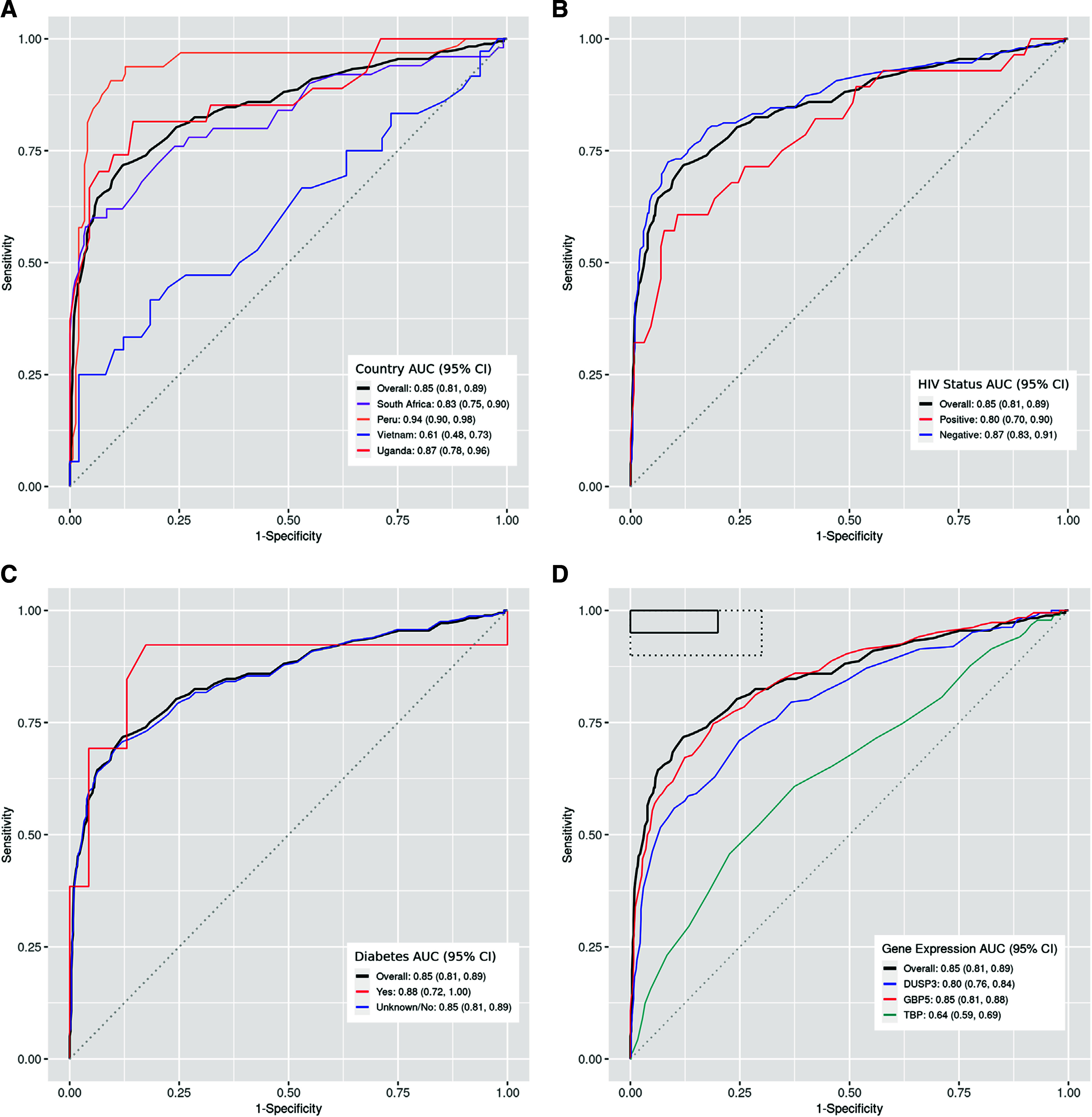
ROC curves for Xpert HR assay tested in capillary blood. **A:** ROC curves for TB score, overall and stratified by country. **B:** ROC curves for TB score, overall and for participants with and without HIV. **C:** ROC curves for TB score, overall and for participants with and without diabetes mellitus; **D:** ROC curves for overall TB score and individual genes. HR = host response; ROC = receiver operating characteristic; AUC = area under the ROC curve.

Xpert HR AUC: Using capillary blood, the overall AUC was estimated to be 0.85 (95% CI: 0.81–0.89) and was highest for participants enrolled in Peru (0.94 [95% CI: 0.90–0.98]) and lowest in Vietnam (0.61 [95% CI: 0.48–0.73]) (Figure 3A). The AUC for people without HIV was higher (0.87 [95% CI: 0.83–0.91]) than that for PLWH (0.80 [95% CI: 0.70–0.90]) (Figure 3B). The AUC for the people with DM was 0.88 (95% CI: 0.72–1.00) (Figure 3C). By individual gene CT value, the AUC for the *GPB5* gene was highest (0.85 [95% CI: 0.81–0.89]), followed by the *DUSP3* gene (0.80 [95% CI: 0.76–0.84]) and the *TBP* gene (0.64 [95% CI: 0.59–0.69]) (Figure 3D).

Sensitivity and specificity at empirically derived TB score optimal cut-off values: The TB score cut-off value that achieved a sensitivity of at least 90% and maximised specificity for capillary blood testing among all analysed participants was −0.90; TB scores at or below this cut-off defined a positive test. Using that cut-off, the overall sensitivity was 91% (95% CI: 86–94) and specificity was 45% (95% CI: 41–49). Sensitivity and specificity were highest in Peru (97% [95% CI: 89–99] and 54% [95% CI: 46–62], respectively) and lowest in Vietnam (83% [95% CI: 68–92] and 27% [95% CI: 16–40], respectively) (Table 2). The NPV was >90% in all countries except for Vietnam, where NPV was 68% (95% CI: 46–85). In PLWH and people without HIV, sensitivities were 93% (95% CI: 76–98) and 91% (95% CI: 85–94), respectively, and specificities were 18% (95% CI: 13–27) and 53% (95% CI: 48–58), respectively (Table 2).

**Table 2.** Diagnostic accuracy of Xpert HR using capillary blood and using the TB score cut-off value that achieved sensitivity of 90% or greater and maximised specificity among all participants.

Participant group	Sensitivity % (n/N) (95% CI)	Specificity % (n/N) (95% CI)	PPV % (n/N) (95% CI)	NPV % (n/N) (95% CI)	AUC (95% CI)
Overall	91 (161/177) (86, 94)	45 (241/539) (41, 49)	35 (161/459) (31, 39)	94 (241/257) (90, 96)	0.85 (0.81, 0.89)
Country					
South Africa^**A**^	90 (45/50) (79, 96)	45 (113/250) (39, 51)	25 (45/182) (19, 32)	96 (113/118) (90, 98)	0.83 (0.75, 0.90)
Uganda^**A**^	89 (24/27) (72, 96)	38 (34/90) (29, 48)	30 (24/80) (21, 41)	92 (34/37) (79, 97)	0.87 (0.78, 0.96)
Peru^**A**^	97 (62/64) (89, 99)	54 (81/150) (46, 62)	47 (62/131) (39, 56)	98 (81/83) (92, 99)	0.94 (0.90, 0.98)
Vietnam^**A**^	83 (30/36) (68, 92)	27 (13/49) (16, 40)	45 (30/66) (34, 57)	68 (13/19) (46, 85)	0.61 (0.48, 0.73)
Age (years)					
<28	93 (54/58) (79, 97)	59 (71/121) (50, 67)	52 (54/104) (43, 60)	95 (71/75) (84, 98)	0.89 (0.83, 0.95)
28–50	91 (79/87) (83, 95)	43 (121/284) (37, 48)	33 (79/242) (27, 39)	94 (121/129) (89, 97)	0.85 (0.80, 0.90)
>50	87 (27/31) (55, 95)	36 (48/132) (29, 45)	24 (27/111) (18, 33)	92 (48/52) (70, 97)	0.78 (0.67, 0.88)
HIV status					
HIV negative	91 (135/149) (85, 94)	53 (217/409) (48, 58)	41 (135/327) (36, 47)	94 (217/231) (90, 96)	0.87 (0.83, 0.91)
HIV positive	93 (26/28) (76, 98)	18 (24/130) (13, 27)	20 (26/132) (14, 27)	92 (24/26) (76, 98)	0.80 (0.70, 0.90)
Sputum smear status^**B**^					
Negative	75 (40/53) (62, 85)	45 (239/533) (41, 49)	13 (40/334) (9, 16)	95 (239/252) (92, 97)	0.63 (0.54, 0.71)
Positive	98 (121/124) (90, 99)	40 (2/5) (12, 84)	98 (121/124) (91, 99)	40 (2/5) (12, 77)	0.92 (0.84, 1.0)
Diabetes					
Yes	92 (12/13) (50, 99)	43 (10/23) (28, 60)	48 (12/25) (33, 63)	91 (10/11) (43, 98)	0.88 (0.72, 1.0)
Unknown	91 (149/164) (85 94)	45 (231/516) (41, 49)	34 (149/434) (30, 39)	94 (231/246) (90, 96)	0.85 (0.81, 0.89)

IQR = interquartile range (25th and 75th percentiles); PPV = positive predictive value; NPV = negative predictive value; AUC = area under the curve; CI = confidence interval.

A Confidence intervals are two-sided stratified Wilson score intervals, except for all country-specific estimates and NPV for sputum smear–positive participants where it was not estimable, in which case the Wilson score confidence interval was substituted.

B One participant who was MRS-negative did not have results from sputum smear microscopy.

### Comparison of Xpert HR results for capillary versus venous blood samples

**Figure 4.**
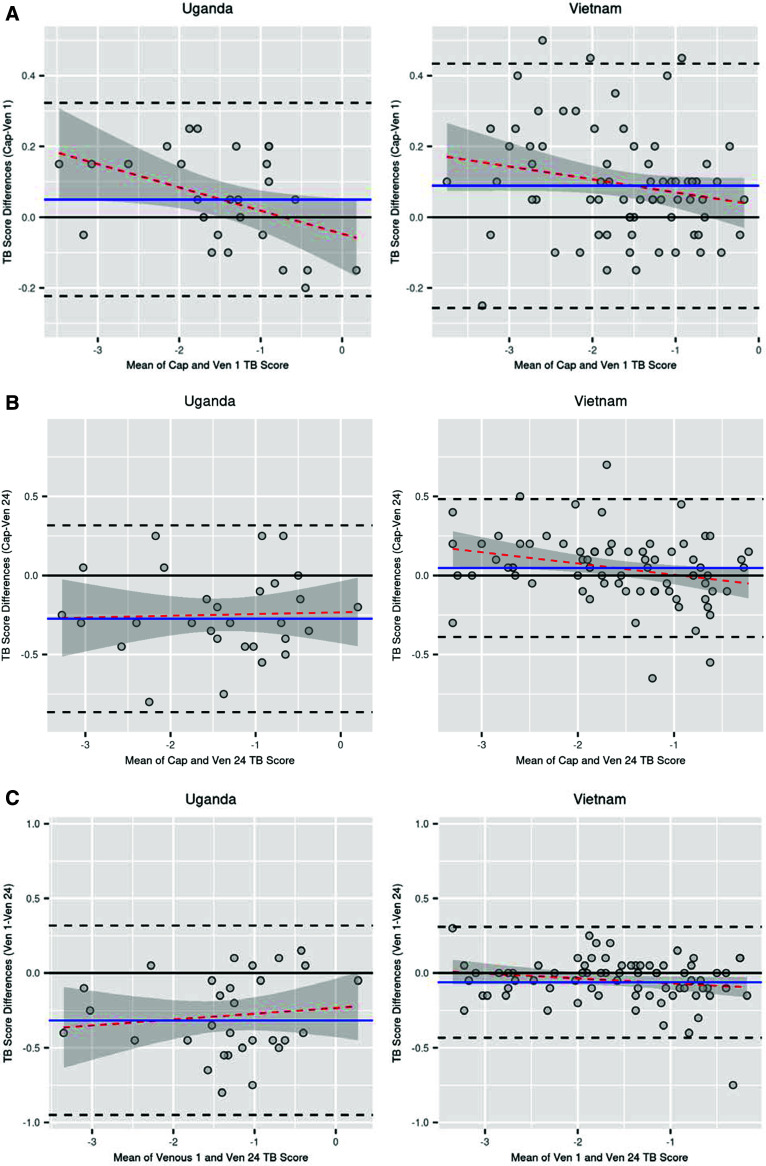
Comparison of Xpert HR assay using capillary blood tested within 1 h and venous blood tested within 1 h and at 24 h of collection. **A:** Bland–Altman analysis results comparing TB scores for capillary blood tested within 1 h and venous blood tested within 1 h of collection by country of enrolment. **B:** Bland–Altman analysis results comparing TB scores for capillary blood tested within 1 h and venous blood tested at 24 h after collection by country of enrolment. **C:** Bland–Altman analysis results comparing TB scores for venous blood tested within 1 h and venous blood tested at 24 h after collection by country of enrolment. The blue line is the bias and black dashed lines are the limit of agreement assuming constant error. The red dashed line is estimated from ordinary linear regression and grey bands are the 95% confidence interval.

Among participants enrolled in Uganda and Vietnam, in the subset who had capillary and venous blood test results, the median (IQR) overall TB scores were −1.5 (−1.8, −0.9) and −1.6 (−2.0, −0.8), respectively. Bland–Altman plots are shown in Figure 4. TB scores for capillary blood were slightly higher than those for venous blood tested at 1 h in Uganda and Vietnam (bias assuming constant error of 0.05 in Uganda and 0.09 in Vietnam). TB scores for capillary blood were lower than those for venous blood tested at 24 h in Uganda (bias assuming constant error of −0.27), while the TB scores for capillary blood were slightly higher than those for venous blood tested at 24 h in Vietnam (bias assuming constant error of 0.05). Passing and Bablok regression analysis of CT values for pairs of samples resulted in estimates and 95% CIs for intercepts and slopes for *DUSP3* and *GBP5* genes that included the null values corresponding to no difference (0 and 1, respectively) but excluded the null values for the *TBP* gene, which suggests that the differences in TB scores between capillary and venous samples are due to differences in CT values for the *TBP* gene (Supplementary Data Figure S1 and Supplementary Data Table S2).

## DISCUSSION

In this prospective study conducted in four TB-endemic settings, we evaluated the performance of the Xpert HR assay for detecting pulmonary TB in symptomatic adults against an MRS. For capillary blood, Xpert HR sensitivity was 91% and specificity was 45% when using an Xpert HR TB score threshold optimised to meet the TPP for a TB triage test minimal sensitivity recommendation while maximising specificity for the overall group. Performance was comparable in Uganda, South Africa, and Peru but was lower in Vietnam. Vietnam was a notable outlier in our study, with lower AUC, sensitivity, and specificity point estimates than those of the other countries. This was not driven by HIV infection, as no participants living with HIV were enrolled in Vietnam. We and others found an inverse association of TB score with sputum smear microscopy results, consistent with the hypothesis that transcriptomic signatures are a proxy for TB disease severity. Among MRS+ participants in Vietnam, low sputum bacillary burden as assessed by smear microscopy and Xpert MTB/RIF Ultra and low prevalence levels of fevers, night sweats, and weight loss all support a low TB disease severity that may be reflected in the relatively high median TB score of −1.6. Additionally, there may be other prevalent diseases, co-infection patterns, environmental factors, and genotypic or phenotypic differences across populations that influence the expression of the tested genes. Existing TB gene signatures were predominantly derived from African datasets, and thresholds or signatures may need to be optimised for use in different populations or geographies. Our findings are aligned with those of Gupta-Wright et al.^16^ and Sutherland et al.,^9^ who also reported site-specific variability, such as a lower specificity in study participants from Vietnam compared with other clinical sites. These observations suggest that variation in disease burden, as reflected by smear status and Xpert Ultra semi-quantitative categories, likely contributes to the differences in test performance seen across studies. For example, studies in which ≥90% of participants had ‘Low’ or higher Xpert results reported higher sensitivity,^9^ whereas cohorts with more participants with very low or trace results showed lower performance^16^ and did not meet the WHO TPP for a TB triage test.

In pre-specified analyses, AUC point estimates and sensitivity did not differ by HIV status, but specificity was only 18% among PLWH versus 53% among people without HIV. HIV drives anti-viral type-1 interferon host responses that underlie TB transcriptomic signatures, and this may be the potential biologic basis for our finding.^17^ Acknowledging that a specificity of 18% is well below the target for a TB triage test, practical implications would be influenced by HIV prevalence.

We found variation in TB scores by specimen type and storage. Passing and Bablok regression analysis suggested that differences in *TBP* CT values were the source of the variation. Accordingly, TB score positivity thresholds optimised for capillary blood, the intended specimen type, should not be applied to venous blood specimens.

Our findings highlight the potential for integrating gene-level analysis into TB diagnostics. Among the genes evaluated, AUC for the *GBP5* gene expression was indistinguishable from the three-gene AUC, as has been observed by others.^9^ This means that measuring GBP5 expression alone may be sufficient and more affordable assays.

Strengths of our study include a robust MRS, multisite cohort, comorbidities such as HIV and DM, large sample size with relatively precise accuracy estimates, and standardisation of procedures. However, we acknowledge some limitations. Our findings may not be generalisable to the entire population of people in whom TB testing is indicated, as we required that participants be able to expectorate sputum, we did not include people with non-pulmonary TB, and we did not undertake the longitudinal participant follow-up required to make a clinical diagnosis of TB in those whose who were negative on initial testing. In addition, we were unable to systematically confirm alternative diagnoses, such as pneumonia, malignancies, or other inflammatory conditions, due to resource constraints, which may contribute to unmeasured confounding. Lastly, country-specific factors such as population demographics, host response, and epigenetics likely influenced our results, underscoring the importance of site-specific validation.

## CONCLUSION

The Xpert HR TB assay demonstrated promising sensitivity overall and in PLWH, although its specificity did not meet the minimum TPP for a TB triage test. In its current form, Xpert HR cannot replace sensitive nucleic acid amplification diagnostic tests in sputum-productive individuals. The role of Xpert HR in the detection of subclinical TB and in people who cannot expectorate sputum remains to be defined.

## Supplementary Material


