# Supplementary material for: Diagnostic accuracy of the Cepheid MTB host response assay for the detection of pulmonary TB
Source: IJTLD Open. 2025 Nov 12;2(11):646–54. doi: 10.5588/ijtldopen.25.0380 (PMC12617089; doi:10.5588/ijtldopen.25.0380)
Supplement: Supplementary file 1 [file ijtldopen25-0380_supplementarydata1.pdf]

Supplementary Table 1. Xpert HR TB scores for participants with a valid score using capillary blood, overall and by MRS status

Characteristic	Xpert HR TB Score, median (IQR)		
	**Overall**	**MRS positive**	**MRS negative**
**All participants**	−1.2 (−2.0, −0.8)	−2.7 (−3.4, −1.7)	−1.0 (−1.5, −0.7)
**Country**			
Peru	−1.2 (−2.5, −0.7)	−3.1 (−3.5, −2.4)	−0.9 (−1.3, −0.6)
South Africa	−1.1 (−1.7, −0.7)	−2.8 (−3.3, −1.6)	−1.0 (−1.5, −0.6)
Uganda	−1.3 (−2.1, −0.8)	−3.0 (−3.5, −1.9)	−1.1 (−1.7, −0.7)
Vietnam	−1.6 (−2.1, −1.0)	−1.6 (−2.6, −1.1)	−1.5 (−1.9, −0.8)
**Sex**			
Male	−1.2 (−2.3, −0.7)	−2.8 (−3.4, −1.9)	−1.0 (−1.5, −0.6)
Female	−1.2 (−1.8, −0.8)	−2.6 (−3.1, −1.6)	−1.0 (−1.5, −0.7)
**Diabetes**			
No	−1.2 (−1.9, −0.8)	−2.7 (−3.4, −1.7)	−1.0 (−1.5, −0.6)
Yes	−1.4 (−2.3, −0.8)	−2.4 (−3.2, −2.0)	−1.1 (−1.5, −0.7)
**HIV status**			
Negative	−1.0 (−1.9, −0.7)	−2.8 (−3.4, −1.7)	−0.9 (−1.3, −0.6)
Positive	−1.6 (−2.0, −1.1)	−2.6 (−3.1, −1.7)	−1.5 (−1.8, −1.0)
**Sputum smear status** †			n/a
Negative	−1.0 (−1.6, −0.7)	−1.3 (−1.9, −0.9)	
Scanty	−2.6 (−3.2, −2.0)	−3.0 (−3.2, −2.2)	
1+	−2.9 (−3.6, −2.4)	−3.0 (−3.7, −2.4)	
2+	−3.1 (−3.5, −2.8)	−3.1 (−3.5, −2.8)	
3+	−3.2 (−3.5, −2.7)	−3.2 (−3.5, −2.7)	
**Sputum Xpert MTB/RIF Ultra semiquantitative result**			n/a
Negative	−1.0 (−1.5, −0.7)	−1.3 (−1.9, −0.9)	
Trace	−1.1 (−1.7, −0.9)	−1.1 (−1.7, −0.9)	
Very Low	−1.3 (−1.6, −0.9)	−1.3 (−1.6, −0.9)	
Low	−2.5 (−3.2, −1.7)	−2.5 (−3.2, −1.7)	
Medium	−3.1 (−3.5, −2.3)	−3.1 (−3.5, −2.3)	
High	−3.1 (−3.5, −2.7)	−3.1 (−3.5, −2.7)	

Abbreviations: TB: tuberculosis, MRS: microbiological reference standard, HIV: human immunodeficiency virus, IQR interquartile range, n/a: not applicable.

†Five participants classified as MRS negative were sputum smear positive and 1 participant did not have a smear status and were excluded from MRS negative column but are represented in the Overall column.

Supplementary Figure 1

A. Passing and Bablok regression comparing capillary and venous 1 hour sample gene CT values

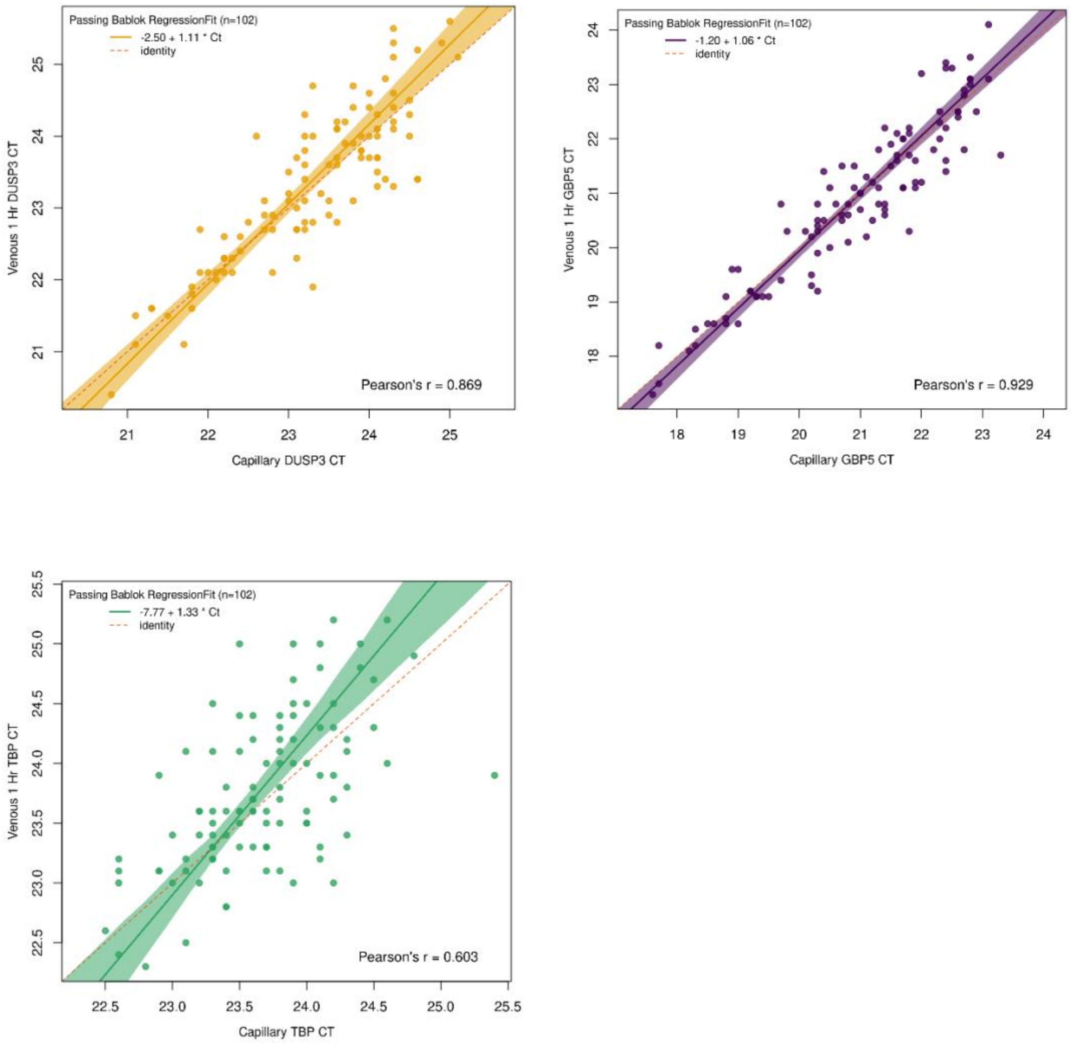

The dashed line is where CT values for capillary and venous 1-hour samples are identical. The solid line and shaded area are the estimated regression line and 95% confidence interval.

B. Passing and Bablok regression comparing capillary and venous 24-hour sample genes CT values

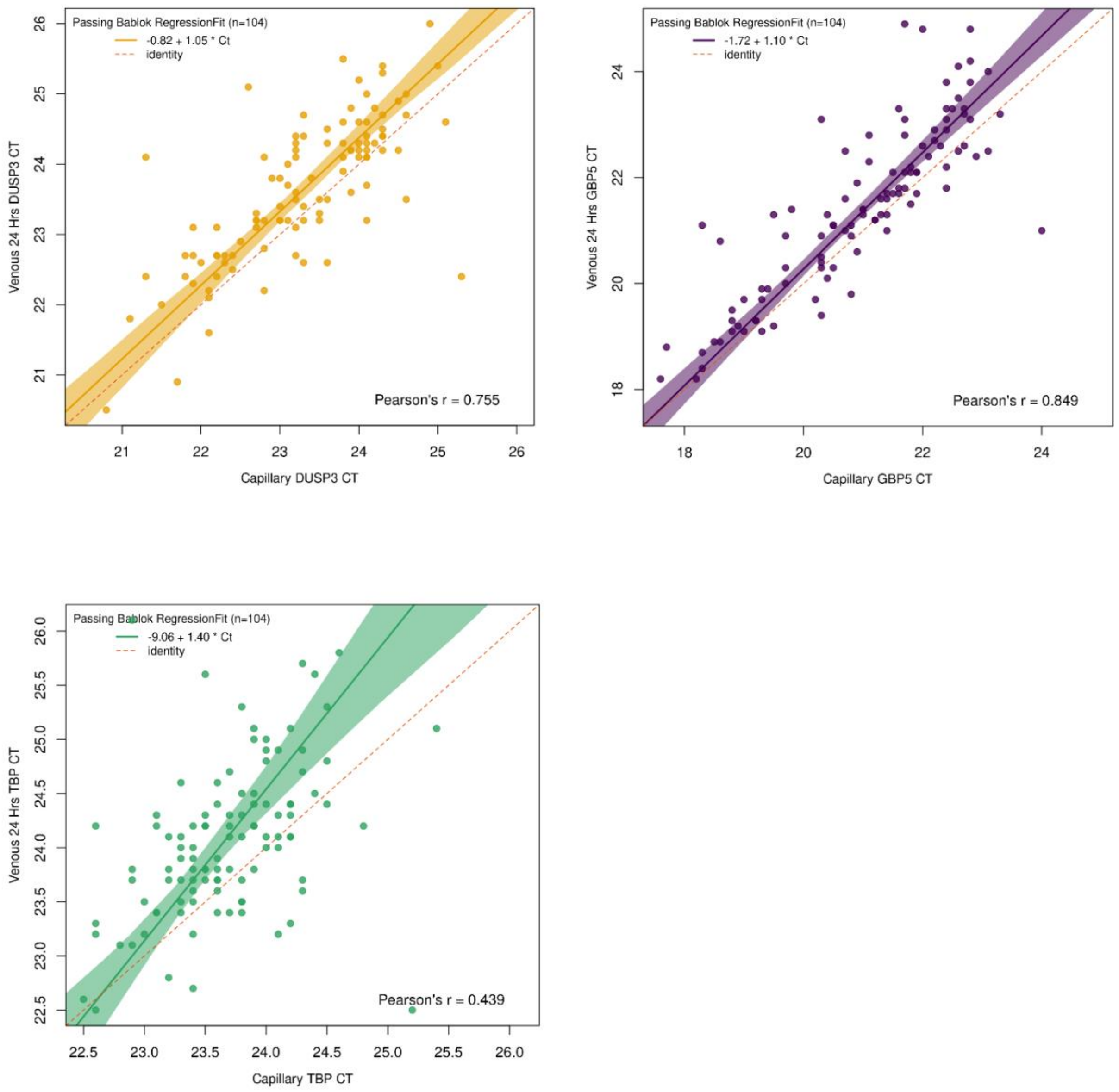

The dashed line is where CT values for capillary and venous 24-hour samples are identical. The solid line and shaded area are the estimated regression line and 95% confidence interval.

C. Passing and Bablok regression comparing venous 1-hour sample and venous 24-hour sample genes CT values

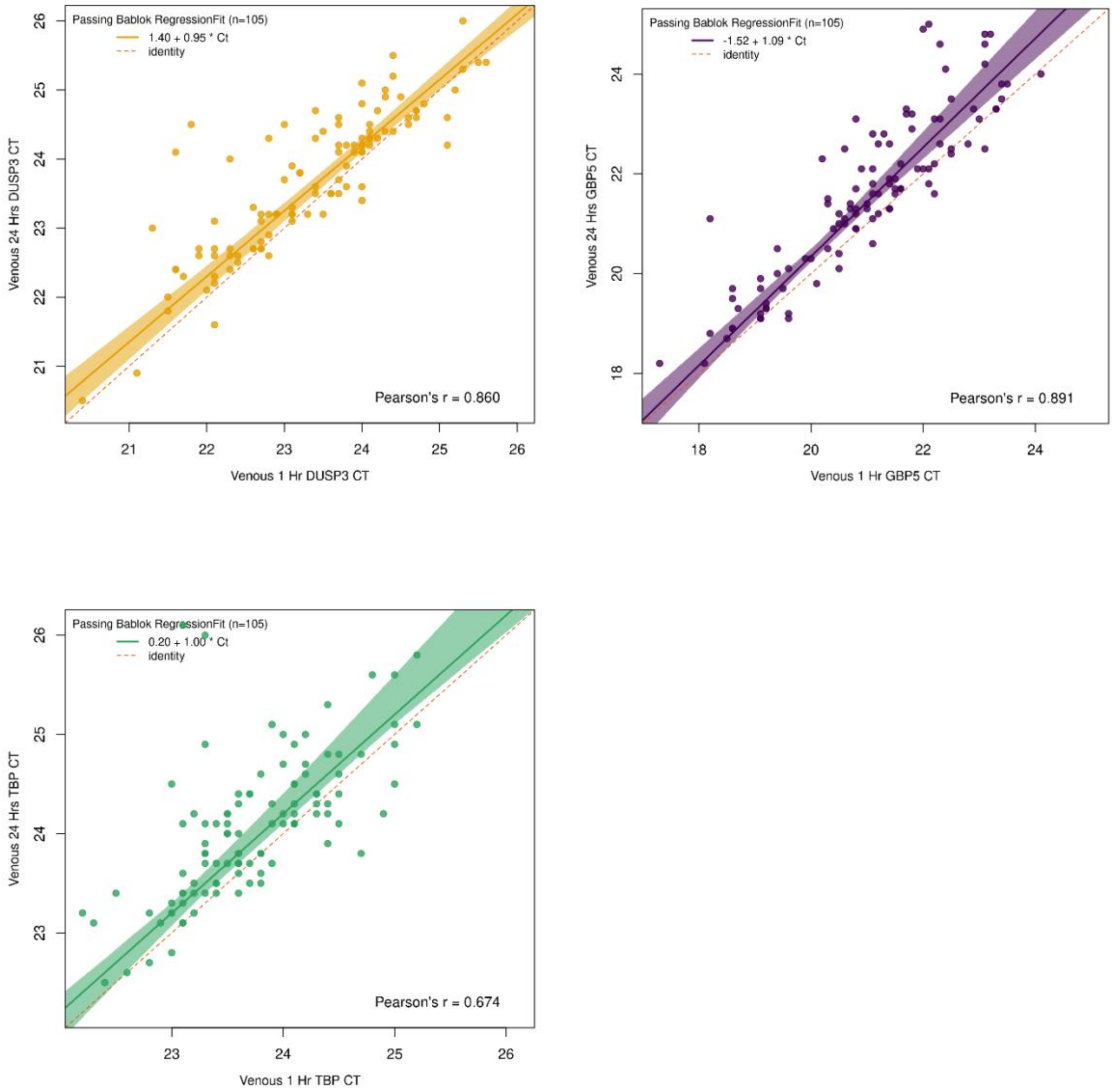

The dashed line is where CT values for venous 1-hour and venous 24-hour samples are identical. The solid line and shaded area are the estimated regression line and 95% confidence interval.

Supplemental Table 2: Summary of Passing and Bablok regression results on individual gene CT values

X-variable	Y-variable	*DUSP3* Intercept (95% CI)	*DUSP3* Slope (95% CI)	*GBP5* Intercept (95% CI)	*GBP5* Slope (95% CI)	*TBP* Intercept (95% CI)	*TBP* Slope (95% CI)
Capillary	Venous 1-hr	−2.50 (−5.13, 0.05)	1.11 (1.00, 1.23)	−1.20 (−2.76, 0.00)	1.06 (1.00, 1.13)	−7.77 (−15.6, −1.47) *	1.33 (1.07, 1.67) *
Capillary	Venous 24-hr	−0.82 (−4.11, 1.52)	1.05 (0.95, 1.19)	−1.72 (−3.92, 0.40)	1.10 (1.00, 1.21)	−9.06 (−16.7, −1.59) *	1.40 (1.08, 1.73) *
Venous 1-hr	Venous 24-hr	1.40 (0.10, 3.50) *	0.95 (0.86, 1.00)	−1.52 (−4.12, 0.41)	1.09 (1.00, 1.22)	0.20 (−4.96, 1.65)	1.00 (0.94, 1.22)

Abbreviations: CI: confidence interval, hr: hour.

A participant was required to have TB scores for all 3 sample type/storage durations (capillary, venous 1-hour, and venous 24-hour) to be included in the analysis; there were 92 participants meeting this criteria.

Using the Passing and Bablok regression we fit the following model to pairs of gene CT values:

Y-variable = Intercept + Slope × X-variable.

An Intercept of 0 and Slope of 1 would be expected if CT values for the pair of sample types or storage durations were identical.

* 95% confidence interval for the Intercept excludes 0.0 or for the Slope excludes 1.0.
